# Commentary: *Propionimicrobium lymphophilum* in urine of children with monosymptomatic nocturnal enuresis

**DOI:** 10.3389/fcimb.2025.1553911

**Published:** 2025-04-07

**Authors:** Susan L. Prescott, Alan C. Logan

**Affiliations:** ^1^ School of Medicine, University of Western Australia, Perth, WA, Australia; ^2^ Department of Family and Community Medicine, University of Maryland School of Medicine, Baltimore, MD, United States; ^3^ Nova Institute for Health, Baltimore, MD, United States

**Keywords:** nocturnal enuresis, propionic acid, neuromicrobiology, legalome, urobiome, biological psychology

## 1 Introduction

Primary monosymptomatic nocturnal enuresis is a neurobiological disorder characterized by frequent nighttime bedwetting in children aged five or older. It is distinguished from secondary enuresis, in which a child has previously experienced at least six months of nighttime dryness, and polysymptomatic enuresis, in which there are clear lower urinary tract symptoms such as urgency and frequent urination. Nocturnal enuresis is relatively common at 5 years old (25% of children) and normally diminishes with age (10% of 7-year-olds and 1 to 2% by age 15) (Walker, 2019). Current treatments, none of which are universally effective, include behavioral modifications, nighttime bed alarms, and pharmacotherapy (e.g., antidepressants and/or desmopressin) (Jørgensen et al., 2023). Nocturnal enuresis is not a trivial condition; it has been associated with comorbid neuropsychiatric disorders, especially those involving hyperactivity and/or problems with impulse control (Hamed et al., 2021).

In a remarkable new study, Nishizaki and colleagues analyzed the urobiome and identified *Propionimicrobium lymphophilum* as a candidate marker for monosymptomatic nocturnal enuresis in children (Nishizaki et al., 2024). The authors are to be commended for their pioneering work, directed at a clinical condition associated with significant suffering in children. Their work joins a separate 2024 study from Japanese investigators that also showed significant urobiome differences in children with untreated nocturnal enuresis (Akagawa et al. (2024). Here is this commentary, we would like to expand on the potential mechanisms offered by Nishizaki and colleagues, including those that can potentially link *Propionimicrobium lymphophilum* to the neuropsychiatric disturbances commonly associated with enuresis.

## 2 Pathogenic mechanisms

In the consideration of possible mechanisms linking *P. lymphophilum* to enuresis, Nishizaki and colleagues briefly speculate that the bacterium releases certain neurotransmitters which, at the central level, interfere with the ability of children to “awaken with urine.” The authors then suggest that ampicillin may be an appropriate therapeutic agent. The origin of the *P. lymphophilum* is not discussed, although it is worth noting that the bacterium is a steroid metabolizing microbe found in the human gut (Li et al., 2022). This is important because urinary tract infections appear to be mediated, at least in part, by gut dysbiosis (Worby et al., 2022; Iqbal et al., 2024). That is, Nishizaki and colleagues may be identifying a downstream consequence of a dysbiotic condition rooted in the gut.

Elaborating on mechanisms, we suggest that dysbiosis, whether at the gut and/or urobiome level, is associated with neurodevelopmental delays, especially those related to inhibitory pathways. In order to make that argument, it is first important to contextualize primary nocturnal enuresis as a problem of inhibition. That is, enuresis, much like the childhood impulse control disorders that often overlap with nocturnal enuresis, appear to be rooted in deficits of inhibitory pathways. For example, research using fMRI in concert with cognitive tasks designed to invoke inhibition shows that children with primary nocturnal enuresis have relative underactivity of prefrontal cortex circuitry—pathways that are critical to inhibitory functions and the suppression of socially inappropriate actions (Lei et al., 2012). In addition to the prefrontal cortex, evidence also suggests that maturation and proper functioning of inhibitory signals in the brain stem are also important to the absence of enuresis (Freitag et al., 2006).

Inhibitory pathways in the lateral area of the pontine micturition center act to inhibit micturition and prevent nocturnal enuresis (Ornitz et al., 1999). Lack of inhibitory signaling in the same general area of the brain is associated with deficiencies in the normal prepulse inhibition of a startle response. Here, prepulse refers to a weak peripheral sensory input (auditory, visual, or tactile) that is presented immediately prior to a sudden and significant stimulus that would otherwise cause a startle response. Normally, the prepulse significantly dampens a startle response, but not in children with nocturnal enuresis (Logan and Lesperance, 2005). Diminished prepulse inhibition of startle has been noted in many neuropsychiatric conditions (Santos-Carrasco and De la Casa, 2023), including those that overlap with nocturnal enuresis (Pole et al., 2022).

With this background, we can return to the work of Nishizaki. The authors note that *P. lymphophilum*, like all propionibacterium, is a significant producer of the short chain fatty acid propionic acid. While gut microbe produced short chain fatty acids are often purported to be universally beneficial, in preclinical research propionic acid has been linked to behaviors that align with neuropsychiatric disorders. Multiple animal studies show that excess propionic acid promotes neuroinflammation and interferes with normal neurodevelopment (Lagod et al., 2024; Alsaqer et al., 2023), and elevated levels of gut-derived propionic acid can lead to neuronal excitotoxicity and lowered levels of the inhibitory neurotransmitter gamma-aminobutyric acid (GABA) (El-Ansary et al., 2018). Most of the research related to propionic acid and impaired neurodevelopment remains in the preclinical realm, although connections between elevated gut-derived propionic acid and autism in humans has been reported (Wang et al., 2012; Xiong et al., 2016).

Of note to Nishizaki’s work, propionic acid has been reported to diminish the prepulse inhibition of startle (Wah et al., 2019), thereby providing a possible direct mechanistic link between *P. lymphophilum* and enuresis. Indeed, microbe-produced propionic acid is emerging as a driver of impaired social and emotional behavior (Huang et al., 2021), which suggests that if the findings of Nishizaki and colleagues are replicated and expanded upon, they might provide a unifying concept wherein microbe-driven propionic acid explains the overlaps between enuresis and neuropsychiatric conditions.

## 3 Future directions

Moving forward it would be helpful if gut microbiome analyses are simultaneously paired with urobiome analysis. It is our contention that the findings of Nishizaki and colleagues can open a door towards a deeper understanding of the neuropsychiatric and neurodevelopmental underpinnings of enuresis. Aggression and other behavioral problems are often assumed to be a psychological consequence of the experience of enduring nocturnal enuresis, rather than being part of central inhibitory deficits that are driven by microbes along the gut-bladder axis. That is, psychology is privileged in the academic discourse, and nocturnal enuresis is erroneously assumed to be a psychiatric disorder (Maternik, 2019). The work of Nishizaki allows for a different vantage point.

An overgrowth of propionic acid-producing bacteria is associated with aggression in animal models (Choi et al., 2018). Indeed, nocturnal enuresis may be an indicator of later risk of later posttraumatic stress disorder (Gurvits et al., 1993) and criminal justice involvement (Koposov et al., 2024). Emergent research involving female prisoners in a carceral facility showed that *Bacteroides* and *Barnesiella* (genera linked with higher gut propionic acid) were significantly higher, and stool propionic acid higher (although not at statistical significance), in women with a history of violence and impulse control issues (Langmajerová et al., 2025). Researchers seeking to expand upon the work of Nishizaki and colleagues should consider a holistic picture of the gut-bladder-microbiome axis and how it intersects with diverse neuropsychiatric disorders. Advances in the research should overcome the limitations of 16S rRNA sequencing, as used by Nishizaki, and include advanced techniques like shotgun metagenomics, as well as multi-omics and polygenic investigations. There is also a need to replicate the work of Nishizaki and colleagues in diverse populations; their work involved Japanese children and there was no mention of socioeconomic variables in the relatively small sample (25 children with monosymptomatic nocturnal enuresis and 17 controls).

While antimicrobial agents such as the ampicillin recommended by Nishizaki may have therapeutic value, they are not without adverse events, including the promotion of gut dysbiosis. Here it is worth noting that a recent fecal transplant study showing that donor fecal material from human infants exposed to antibiotics in early life led to increased aggression in recipient animals (vs. animals receiving transplants from unexposed infants) (Uzan-Yulzari et al., 2024). On the other hand, preclinical models of neurodevelopmental delay and neuropsychiatric conditions indicate that the application of select microbes can help remedy diminished prepulse inhibition (Hsiao et al., 2013; Liao et al., 2019). Given recent human research highlighting the potential value of probiotics in impulse control (Montazeri et al. (2025; Levy Schwartz et al., 2024), trials in childhood enuresis seem worthwhile.

## 4 Conclusion

Notwithstanding the design limitations, Nishizaki and colleagues made an important advance in the study of nocturnal enuresis. In the discussion of their findings, the authors briefly touched upon some potential mechanisms linking their discovery—a significantly higher urinary presence of *Propionimicrobium lymphophilum*—to analyze the urobiome in urine samples obtained from the two groups of children. Given the relationship between propionic acid-producing bacteria and neurobehavioral disturbance, as well as impaired inhibition (Al Suhaibani et al., 2021; El-Ansary et al., 2012), Nishizaki and colleagues may have opened the door to multiple lines of inquiry. Our commentary is not intended to suggest that propionic acid is an explanatory biochemical providing clear and obvious mechanistic links between enuresis and impulse control disorders. However, the work of Nishizaki brings neuromicrobiology into discussions of a disorder that is all too often trivialized, and raises many research-based questions for transdisciplinary collaborators.
